# Comparison of the novel VieScope with conventional and video laryngoscope in a difficult airway scenario – a randomized, controlled simulation trial

**DOI:** 10.1186/s12873-021-00484-6

**Published:** 2021-07-30

**Authors:** Hannes Ecker, Simone Kolvenbach, Sebastian Stranz, Holger Herff, Wolfgang A. Wetsch

**Affiliations:** University of Cologne, Medical Faculty, and University Hospital Cologne, Department of Anaesthesiology and Intensive Care Medicine, Kerpener Str. 62, 50937 Cologne, Germany

**Keywords:** Endotracheal intubation, Difficult airway management, Difficult airway, Video-laryngoscopy, VieScope

## Abstract

**Background:**

Endotracheal intubation continues to be the gold standard for securing the airway in emergency situations. Difficult intubation is still a dreadful situation when securing the airway.

**Objective:**

To compare VieScope with Glidescope and conventional Macintosh laryngoscopy (MAC) in a simulated difficult airway situation.

**Methods:**

In this randomized controlled simulation trial, 35 anesthesiologists performed endotracheal intubation using VieScope, GlideScope and MAC in a randomized order on a certified airway manikin with difficult airway.

**Results:**

For the primary endpoint of correct tube position, no statistical difference was found (*p* = 0.137). Time until intubation for GlideScope (27.5 ± 20.3 s) and MAC (20.8 ± 8.1 s) were shorter compared to the VieScope (36.3 ± 10.1 s). Time to first ventilation, GlideScope (39.3 ± 21.6 s) and MAC (31.9 ± 9.5 s) were also shorter compared to the VieScope (46.5 ± 12.4 s). There was no difference shown between handling time for VieScope (20.7 ± 7.0 s) and time until intubation with GlideScope or MAC. Participants stated a better Cormack & Lehane Score with VieScope, compared to direct laryngoscopy.

**Conclusion:**

Rate of correct tracheal tube position was comparable between the three devices. Time to intubation and ventilation were shorter with MAC and Glidescope compared to VieScope. It did however show a comparable handling time to video laryngoscopy and MAC. It also did show a better visualization of the airway in the Cormack & Lehane Score compared to MAC.

**Trial registration:**

The study was registered at the German Clinical Trials Register www.drks.de (Identifier: DRKS00024968) on March 31st 2021.

## Introduction

Endotracheal intubation continues to be the gold standard for securing the airway in emergency situations [1–3]. This is usually achieved by direct laryngoscopy, but is increasingly being supplemented by video laryngoscopy - and in some cases even replaced by it [4–9]. Difficult intubation due to anatomical conditions or other situational circumstances is a dreadful situation when securing the airway [10–14], occurring in estimated 1–6% of the cases [15]. There is evidence that endotracheal intubation outside the operating theatre is associated with increased difficulty, increased rate of difficult intubation, lower success rated and higher complication rates [12, 13] This is also associated with an increased risk for consecutive hypoxia, which may – if untreated – become lethal within a very short time. This makes difficult airway situations a great challenge for medical staff, and a potentially life-threatening situation for the patient [12–14].

A novel tool in airway management is the VieScope (Androit Surgical LCCC, Oklahoma City, USA), which can enable a direct line of sight toward the larynx and facilitate endotracheal intubation in a two stage process via a bougie. The VieScope itself was originally designed for deployment in EMS and combat medicine, because of its relatively small logistical effort and immanent ready-for-use-quality.

However, data on VieScope, especially in in-hospital settings, are scarce.

The aim of this study was to compare time and success rates of endotracheal intubation with VieScope in comparison to conventional and video laryngoscope in a randomized, controlled simulation study in a difficult airway scenario.

## Methods

### Ethics approval

The Ethics Committee of the University of Cologne approved the study on 02.02.2021 (ID 20-1475_2; Head: Prof. Dr. Raymond Voltz). The study was conducted in accordance with CONSORT guidelines.

### Study registration

The study was registered at the German Clinical Trials Register www.drks.de (Identifier: DRKS00024968) on March 31st 2021.

### Study design

This study was conducted in April 2021 in the facilities of the University Hospital of Cologne as a randomized controlled manikin trial.

Thirty-five volunteers, all physicians from the Department for Anesthesiology and Intensive Care Medicine at the University Hospital of Cologne, were included after giving written and informed consent.

Inclusion criteria were individuals working as physician in Anesthesia or Critical Care, and age between 18 and 65 years. There were no other exclusion criteria.

### Study protocol

After informed consent, the following demographic and medical background data of the test participants were recorded in pseudonymized form:

1. Gender
2. Age
3. Specialization
4. Medical experience level (years of professional experience)
5. Approximately how many intubations per year?

The participants were then asked to perform endotracheal intubation on a certified airway training manikin (AirSim Advance X, TruCorp Ltd., Lurgan, Northern Ireland) using either VieScope (Androit Surgical LCCC, Oklahoma City, USA), video-laryngoscopy (GlideScope, Verathon Medical, Bothell, USA) or conventional laryngoscopy (MAC - Macintosh-Blade Size 3, Heine, Herrsching, Germany) in randomized order.

In all scenarios, the tongue of the manikin was inflated to a pressure of 35 mbar (as recommended by the manufacturer) and a cervical collar (Stifneck, regular, Laerdal, Stavanger, Norway) was applied to simulate a difficult airway situation.

The order of the devices was randomized using sealed opaque envelopes. A blocked randomization strategy was generated using an online tool (Sealed Envelope Ltd. 2020: www.sealedenvelope.com/simple-randomiser/v1/lists [Accessed 6 Oct 2020]).

Time measurements started with the beginning of airway measures (taking up the laryngoscope) and ended with the first ventilation (using a resuscitation bag).

A determination of the best Cormack & Lehane score was asked verbally and protocolled after every simulation for each airway management device.

The following data was recorded for all three devices in pseudonymized form:

1. Tube position: tracheal vs. esophageal (primary endpoint)
2. Time until intubation (secondary endpoint)
3. Time until first correct ventilation (secondary endpoint)
4. Handling time/Time until bougie - VieScope only (secondary endpoint)
5. Best Cormack & Lehane score (secondary endpoint)

Each simulation was terminated after successful intubation or after 5 min, at which irreversible hypoxia of the patient must be assumed.

### Materials

**Fig. 1:**
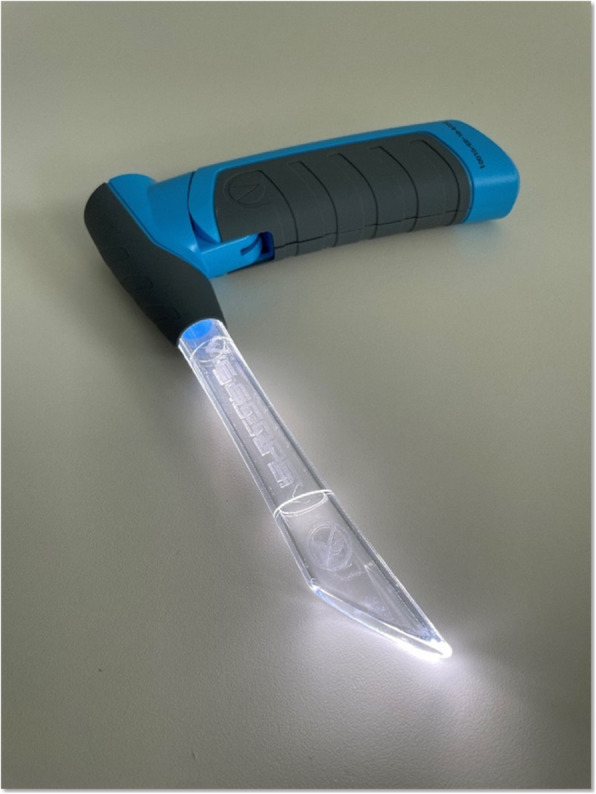
VieScope. VieScope, Adult Size (Androit Surgical LCCC, Oklahoma City, USA), in activated state with illuminated blade

**Fig. 2:**
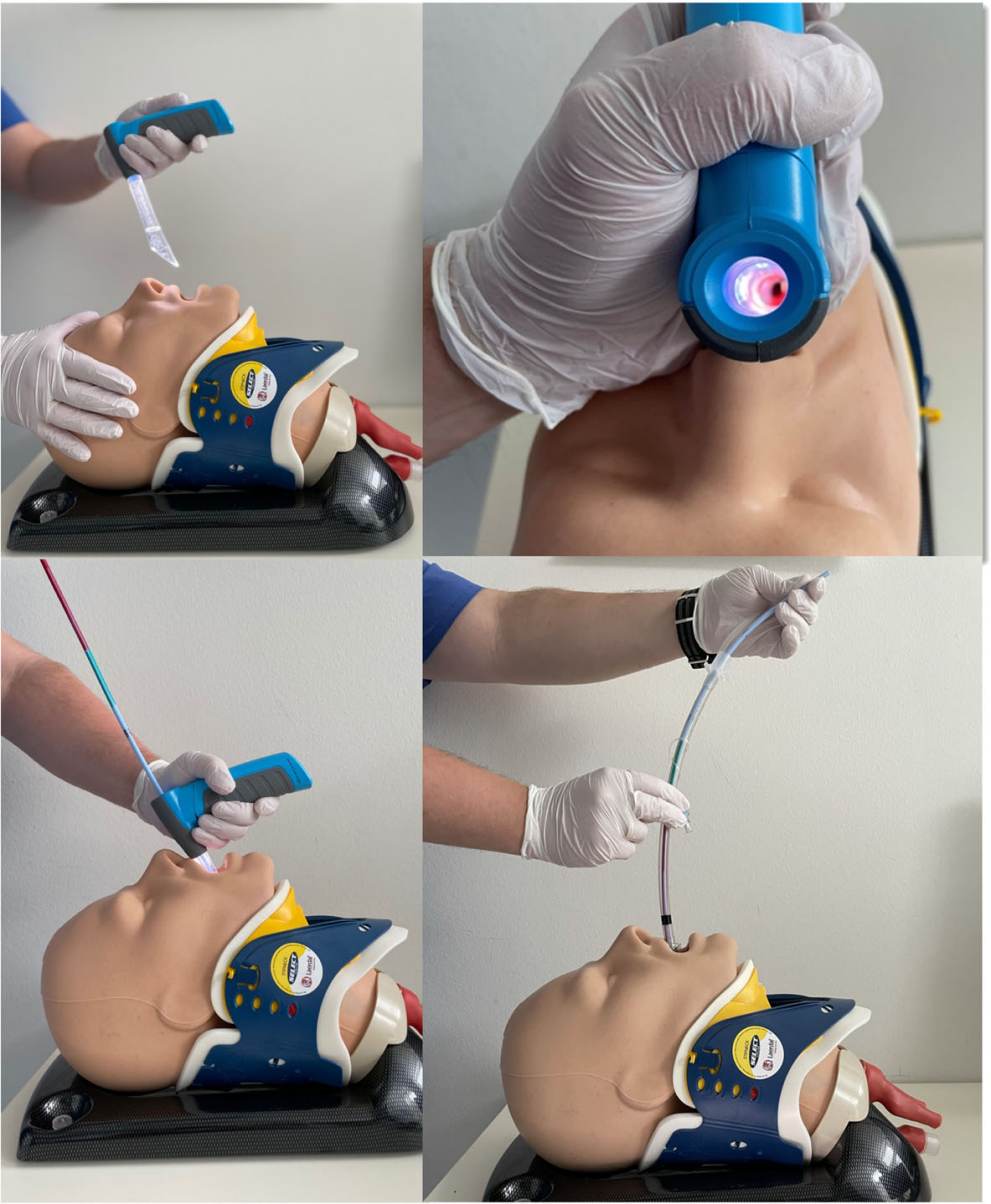
**a**-**d**: Intubation using the VieScope. **a:** VieScope features a straight Miller-shaped laryngoscope blade, which is transparent and illuminated, to allow direct visualization of the vocal cords. **b:** VieScope in place, with visualization of the vocal cords. **c:** Insertion of the bougie into the trachea; after successful insertion, the VieScope can be removed, leaving the bougie in place. **d:** Insertion of the tracheal tube using the bougie as guidance.

For this study, the novel VieScope (VieScope “Training Demo”, Adult Size, Androit Surgical LCCC, Oklahoma City, USA) was used. It consists of a transparent circular straight tube (comparable to a Miller laryngoscope blade), which is illuminated by light emitting diodes (LEDs), and a battery handle. The VieScope is a standalone device, battery powered, and disposable after a single use (Fig. 1). As the scope itself has a straight, Miller-shaped blade, it facilitates a direct and straight view of the glottis, but does not allow direct intubation due to a small inner diameter. Instead, it requires the insertion of a bougie once sight to the vocal cords is achieved. Afterwards, an endotracheal tube can be passed into the trachea over the bougie, which then can be removed (Fig. 2). As bougie, the VOIR Tactical Bougie (Androit Surgical LCCC, Oklahoma City, USA) was used in the attempts with VieScope, whereas the rigid GlideRite stylet (Verathon Inc., Bothell, WA, USA) was used for intubation with GlideScope (Verathon Inc., Bothell, WA, USA).

For conventional Laryngoscopy (MAC) a Macintosh-Blade (Heine, Herrsching, Germany) Size 3 was used, a stylet (Portex Stylet medium, Smiths Medical, Ashford Kent, GB) was inserted into the endotracheal tube beforehand.

### Statistical analysis

Statistical computations were carried out using IBM SPSS Statistics (Version 25; IBM Inc., Armonk, NY, USA).

Since the physicians did not have any experience with the novel VieScope, and there was no published data on success rates, we estimated a difference in first attempt intubation success of 25% compared to the Glide Scope (which was familiar to participants), which has a success rate of 95%. Based on this we calculated that the required sample size in order to achieve 80% power at a significance level of 5% would be 35 participants to detect a difference.

For the comparison of the primary endpoint “Tube position” with GlideScope, MAC and VieScope a Chi-Square-Test was performed.

Secondary endpoints: “time to intubation”, “time to ventilation” and “handling time”, were analyzed after testing for normal distribution (Shapiro-Wilk) and equal variance test (Brown-Forsythe), using a one-way analysis of variance (ANOVA) for repeated measurements to determine the overall statistical significance between the groups. This was then followed by post hoc Student-Newman-Keuls Test method for pairwise multiple comparisons between two groups; *p* < 0.05 was considered as being significant.

## Results

### Demographic and background data

Thirty-five participants, all staff anesthesiologists, were recruited for this study. Of these 35, 18 were female and 17 were male. The mean age was 35 years (range 27 to 47 years), with an experience in the job of < 1 year in *n* = 2 (6%), 1 to 3 years in *n* = 7 (20%), 3 to 6 years in *n* = 8 (23%) and > 6 years in *n* = 18 (51%). The average annual tracheal intubation per participant were less than 50/year in *n* = 3 (9%), 50 to 100 intubations in *n* = 11 (31%), 100 to 200 intubations in n = 7 (20%), and more than 200 intubations in *n* = 14 (40%).

Of all 35 participants, all *n* = 35 (100%) had experience with MAC (in the airway manikin and in the clinical setting), *n* = 34 (97%) had prior experience in the use of the GlideScope (in the airway manikin and in the clinical setting), while only *n* = 10 (29%) had prior experience in the use of the VieScope (only in the airway manikin, as this new device was not routinely used in clinical praxis at the time of the study).

**Fig. 3:**
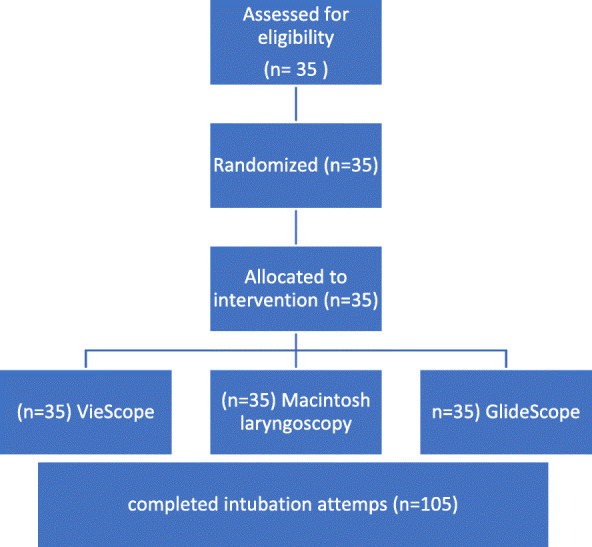
Flow chart. Each participant performed intubation in all settings in a randomized controlled order. There were no drop-outs

In randomized order, each participant performed three endotracheal Intubation attempts in total, one with the GlideScope, one with VieScope, and one with a MAC laryngoscope. Thus, 105 data sets were acquired (Fig. 3).

### Tube position - endotracheal vs. esophageal

For the primary endpoint “tube position in the first attempt” in a difficult airway setting, 35 (100%) had a correct endotracheal position with the GlideScope, 32 (91%) with the MAC and 31 (89%) had correct endotracheal position with the VieScope (*p* = 0.137).

### Time to intubation

The secondary endpoint “time until successful endotracheal intubation” in the difficult airway setting showed a mean ± SD time of 27.5 ± 20.3 s for the use of the GlideScope versus 20.8 ± 8.1 s for the use the MAC versus 36.3 ± 10.1 s for the use of VieScope.

An all pairwise multiple comparison procedures showed that both the GlideScope and the MAC had a shorter time until intubation, compared to the VieScope (*p* < 0.001 both). Time until endotracheal intubation for MAC was shorter compared to the GlideScope (*p* = 0.045).

### Time to first ventilation

Time until first ventilation showed a mean time of 39.3 ± 21.6 s for the use of GlideScope and 31.9 ± 9.5 s for MAC and 46.5 ± 12.4 s in VieScope group.

MAC had a shorter time until ventilation, compared to the VieScope (*p* < 0.001). GlideScope compared to Viescope showed a shorter time until ventilation (*p* < 0.001). Time until ventilation compared between Glide Scope versus MAC also showed a shorter duration (*p* = 0.046).

### Handling-time / time until bougie

Time until bougie placement for VieScope showed a mean time of 20.7 ± 7.0 s compared to time until intubation with GlideScope 27.5 ± 20.3 s and MAC 20.8 ± 8.1 s.

There was no difference when comparing “Time until bougie – placement” with VieScope and “Time until Intubation” with GlideScope or MAC (*p* = 0.527).

### Best Cormack & Lehane scoring

The comparison of the best Cormack & Lehane scoring given by the participants after every intubation with each device, showed that MAC had a mean (±SD) score of 1.6 (± 0,7), while participants using VieScope reported a better score of 1.2 (± 0,4) (*p* < 0.001).

## Discussion

This study investigated the novel VieScope in a randomized controlled simulation trail for difficult airway. Thirty-five anesthetists performed endotracheal intubation using VieScope, GlideScope and Macintosh laryngoscopy (MAC) in a randomized order on a certified airway manikin with difficult airway, generated with an inflated tongue and the application of a cervical collar.

For the primary endpoint of correct tube position, no statistical difference was found between the groups. Regarding the secondary endpoints, time until intubation and first ventilation, GlideScope and MAC had a significantly shorter time compared to the VieScope. There was no significant difference shown between handling time for VieScope and time until intubation with GlideScope or MAC.

Participants stated a better Cormack & Lehane Score with VieScope, compared to direct laryngoscopy.

Although VieScope did not perform better in this study in regard to intubation and ventilation time, it had some relative handicaps which must be considered when comparing it to the other airway devices:

Handicap one was that clinicians in this study were all highly experienced in the use of video laryngoscopy with the GlideScope, since it is the primary and most-used tool in the participating hospital for difficult airway management.

The design of the VieScope itself poses an additional handicap, as it requires a secondary procedural step: the mandatory use of a bougie, over which the tube must be introduces. This additional step takes some time that is not needed when using the other devices. Comparing this device to others, which allow direct intubation, is therefore difficult and this must be kept in mind.

In an effort to balance this out, we created the above-mentioned metric for handling time or time until bougie placement for the VieScope and compared it to time until intubation for GlideScope and MAC. Comparing these times did not show a difference – however, an airway is not secured by the introduction of a bougie alone, and the following step is mandatory.

Unaffected by this, participants stated more often a better Cormack & Lehane (C/L) score with VieScope, compared to MAC, which can be seen as a proof of better visualization provided by VieScope. Unfortunately, as C/L score refers to structures seen in direct laryngoscopy, and GlideScope is an indirect laryngoscope, so we did not report C/L scores for Glidescope and it is not available as comparator.

As it is a new tool, data on VieScope in general is scarce, and we could not find data on it use in the in-hospital setting. One study investigated VieScope’s feasibility in different difficult airways in a simulated pediatric patient with 55 paramedics, which makes a comparison to the adult model difficult [16].

Another study by the same group performed with 42 paramedics in a simulation trial compared VieScope with MAC in two different difficult airway settings (tongue edema or cervical inline stabilization). Here, VieScope had a shorter time until intubation (30.5 s vs. MAC 55.0 s) and higher success rate in first intubation attempt (95.2% vs. MAC 64.3%) [17].

In our study, the mean intubation time was 36.3 s and success rate on first attempt was 89%, also the mean time for use of MAC was 20.8 s with a success rate of 91%. Differences in these results can be explained by the fact that our study recruited anesthesiologist with a very high skill set in regard of airway management, especially with the MAC, and that the difficult airway in our study was a combination of tongue edema and cervical inline stabilization.

As both studies have in common that participants did not have extended experience in the use of VieScope, it can be argued that VieScope seems to be a tool with acceptable success rates even for novice users.

### Limitations

We tested VieScope in an artificial adult simulation scenario. Of course, a manikin can hardly completely generate physiological situation of a patient and simulation studies are not able to fully assess human factor elements (stress, cognitive overload etc.) of the clinicians. However, we rely on simulations trials, as testing a new device always requires a degree of standardized laboratory testbed, before it can be evaluated in daily patient care. Additionally, emergency situations, such as “difficult airway”, are normally not predictably and study enrollment is normally only retrospectively feasible.

## Conclusion

Rate of correct tracheal tube position was comparable between the three devices. Time to intubation and ventilation were shorter with MAC and Glidescope compared to VieScope. It did however show a comparable handling time to video laryngoscopy and MAC. It also did show a better visualization of the airway in the Cormack & Lehane Score compared to MAC.

## Data Availability

All data are included in the manuscript. The original datasets analysed during the current study are available from the corresponding author on reasonable request.
